# How patients experience discussing couple relationship problems with GPs: an interview study

**DOI:** 10.3399/BJGPO.2024.0044

**Published:** 2024-10-16

**Authors:** Siri Dalsmo Berge, Mette Brekke, Eivind Meland, Thomas Mildestvedt

**Affiliations:** 1 Department of Global Public Health and Primary Care, University of Bergen, Bergen, Norway; 2 Department of General Practice, Institute of Health and Society, University of Oslo, Oslo, Norway; 3 Department of Global Public Health and Primary Care, University of Bergen, Bergen, Norway; 4 Department of Global Public Health and Primary Care, University of Bergen, Bergen, Norway

**Keywords:** Family relationships, Consultation, Qualitative research, General practitioners, Primary healthcare

## Abstract

**Background:**

Couple relationship satisfaction is related to good physical health, good mental health, and longevity. Many patients have discussed or wish to discuss their couple relationship with their GP and look for personalised care and support when discussing topics they perceive as sensitive.

**Aim:**

To explore patient experiences of discussing couple relationship problems in GP consultations.

**Design & setting:**

Qualitative study employing semi-structured interviews with patients from general practice in Norway.

**Method:**

Individual interviews with 18 patients who had discussed their couple relationship with their GP. Participants were recruited through both social media and traditional media, and all interviews were digitally recorded. The purposive sample comprised 13 women and five men, representing diverse age groups, backgrounds, and relationship problems. All participants identified as heterosexual. We analysed interview data thematically using systematic text condensation.

**Results:**

Three main themes emerged: 1) GPs in a facilitating role, not on an assembly line; 2) navigating the 'elephant in the room'; and 3) GPs as biomedically competent life witnesses. GP continuity was vital in fostering the trust required to discuss sensitive topics, such as relationship issues. Participants valued a biopsychosocial approach that incorporated knowledge of close relationships into medical consultations. They appreciated both GP support and constructive challenges that prompted them to take responsibility for relationship improvements.

**Conclusion:**

Patients value their GPs’ holistic, supportive, and direct approach in addressing couple relationship problems, although they perceive that GPs do not always have sufficient time. They welcome relevant challenges that can drive positive change.

## How this fits in

In a previous study, we found that one out of four patients had raised couple relationship problems during GP consultations. In this study, we explore patient experiences of such consultations. Through individual, semi-structured interviews, participants emphasised the importance of continuity in establishing trust as a foundation for discussing these sensitive issues. They both appreciated and expected GPs to adopt a biopsychosocial approach in addressing their concerns. The patients not only valued receiving support during challenging times, but also welcomed being appropriately challenged to effect positive changes in their relationships.

## Introduction

Couple relationship satisfaction is related to physical health, mental health, and longevity,^1,2^ and is more important for life quality than job satisfaction and friendships.^3^ Children’s health is also influenced by their parents’ relationship quality.^4,5^

Previous studies examining patient experiences of discussing sensitive topics in GP consultations have highlighted the challenges faced by GPs^6^ and the desire for personalised care and support.^7,8^ A positive GP–patient relationship holds therapeutic value.^9^

GPs integrate elements from various psychotherapeutic approaches^10^ to provide counselling to individuals and couples, utilising their professional expertise and personal experiences.^11^ General practice characteristics like continuity of care and familiarity with patient history facilitate therapeutic consultations,^11,12^ while understanding patient experiences helps identify areas requiring attention.^13^

Mental health discussions, including relational and couple issues, feature in one in four GP consultations.^14^ A substantial proportion of psychosocial consultations revolve around family and partner conflicts.^15^

Fewer than half of those experiencing divorce seek marital therapy before separation.^16^ Couples primarily seek help through therapy or education, which, though effective, reach only a limited population.^16,17^ Brief interventions offer an accessible alternative with comparable efficacy to traditional couple relationship education.^18^ The impact of informal help-seeking, like self-help books or internet resources, remains understudied.^16^

In a Norwegian GP survey, 25% of patients discussed couple relationships, with one-third wanting to consult their GP for couple relationship issues, and nearly half hoping for their GP’s interest.^19^ Another study showed similar patient expectations.^20^

However, data on patient experiences and expectations regarding couple relationship issues in GP consultations are lacking. It remains unclear if patients view GPs as counsellors for such issues, and the dynamics of these consultations from the patient’s perspective remain largely unexplored.

Engel argued that health issues are part of a system wherein biological, psychological, and social factors interplay. Physicians, inspired by systems theory, should assess these factors.^21^ Engel highlighted patient responsibility in illness management, emphasising physician support.^22–24^ Since Engel’s pioneering article in 1977, numerous studies have explored chronic stress on health. This includes stress related to relationship issues.^1,25,26^

The aim of this study was to explore patient experiences of discussing couple relationship problems in GP consultations.

## Method

### Study design and recruitment

This was an exploratory qualitative study with a purposive sample of patients from Norway. We used a multi-media approach to distribute an information film about our study with the aim of recruiting participants. This approach included social media posts, GP clinic websites, and information screens in GP waiting rooms.^27^ We encouraged patients to contact the researchers via articles and interviews in local and regional newspapers, as well as through lectures held in local communities. Thereby, the recruitment process aimed for national coverage. Sample size was governed by information power.^28^ Participants received no payment or other benefits for being part of the study. No pre-existing relationship existed between the researchers and the participants.

We aimed for a purposive selection of patients ensuring maximum variation, in terms of gender, age, and location, to make sure the results would be transferable to clinical practice. Of the 22 patients who contacted the first author, we included 18, aged ≥18 years. Only patients who had discussed their couple relationship with their GP were included. Of the four patients not included in the study, three had not engaged in discussions about their couple relationship with their GP, and one dropped out. Experience of couple relationship problems was defined by the participants themselves.

All participants gave their written consent to take part and understood that they could withdraw their consent at any time.

### Data collection

We conducted 18 semi-structured, individual interviews between January 2021 and February 2022. The first author conducted and recorded all the interviews using the digital platform Zoom. Thirteen interviews were held over Zoom, three via telephone, and two in face-to-face meetings at the first author’s GP office. Each interview lasted 30–45 minutes. The authors developed and used a semi-structured, flexible interview guide as shown in Table 1.

**Table 1.** Interview guide used in the individual interviews with patients about their experiences discussing couple relationship problems in GP consultations (2021–2022)

Interview guide
Research project introduction: brief overview
**What is your experience regarding discussing your relationship with your GP?**
Can you provide any specific examples? How did the topic arise in your conversations? What were your objectives when bringing up this topic? What were your expectations from the doctor? How did your GP handle the conversation?
**In what situations do you believe it is pertinent to discuss relationship issues with your GP?**
Could you share examples of specific instances where you found it relevant to discuss your relationship with your GP? Have you ever wanted to address relationship concerns, but your GP did not provide an opportunity? Could you elaborate on what transpired in such cases? What aspects do you find beneficial when meeting with your GP to discuss relationship problems?
**What have been your experiences in discussing relationship problems involving children with your GP?**
Can you provide instances where children were the focus of conversations when discussing relationship issues with your GP? Do you have examples of situations where discussing child custody or childcare became relevant? What role do you think the GP should assume concerning children and relationship problems?
**What role do you believe your GP should play when patients experience relationship problems?**
What are your expectations regarding their involvement? Do you think GPs require enhanced competence in addressing relationship difficulties, and if so, in what areas? What specific contributions do you hope your GP could make in addressing relationship problems?

The interviewer critically examined her own role to prevent influencing participants’ responses. Considering information power,^28^ we collected sufficient data to meaningfully answer the research questions. Our aim was to encompass a broad spectrum of experiences, including instances of violence, parental alienation, sexual problems, among others. We were aware that certain topics could be considered taboo and thus required a respectful approach, both during the recruitment and the interview processes.

### Data analysis

The first author transcribed the interviews verbatim and anonymised the data before the other researchers commenced the analysis. The analysis was undertaken collaboratively by all four authors, employing thematic and explorative techniques via systematic text condensation.^29^ While we did not base our analysis on an existing theoretical framework, the researchers are all GPs who approach their respective practices from a biopsychosocial standpoint, inspired by the pioneering work of Engel and others.^21,22,30^

Initially, we established an open-minded overview of the data to gain a general impression. Subsequently, we organised the data and identified meaning units by mutually agreeing on preliminary themes. We did not use any qualitative data software. The meaning units were categorised systematically into tables according to the code groups. This process was iterative, as we reorganised the meaning units until a consensus on the final categories was reached to describe the data (Table 2). Thereafter, the meaning units were merged and the content of each subgroup was condensed. Finally, we formulated descriptions and concepts by recontextualising the data.

**Table 2.** Categories and subcategories identified as part of the systematic text condensation and most representative quotes from the in-depth interviews with patients about their experiences discussing couple relationship problems with their GP (2021–2022)

Categories	Subcategories	Quotes	Participant characteristics
GPs in a facilitating role, not on an assembly line	Practical follow-up, the GP as a gateway or gatekeeper	*'Yes, the GP is a junction point, or ... a place to go, and then they refer you to other specialists you need*.'	Participant 10, female, aged 61–70 years.
	Partners	*'It is absolutely easier to bring this up in a GP consultation. Going to couples therapy is a very big step … So I think it would have been difficult to get him to come with me to that*.'	Participant 16, female, aged 61–70 years.
	Time squeeze or prioritising time	' *… I think that in twenty years, that is what I am going to remember. That my GP gave me the last appointment for the day because she knew she had extra time then*.'	Participant 14, female, aged 51–60 years.
Navigating the 'elephant in the room'	A direct approach	*' … my GP had a direct approach, challenging the validity of my assessments and questioning my responsibility for the problems …'*	Participant 7, female, aged 51–60 years.
	Engaged and interested GP	*'That they have time and that they make time, that they are connected, that they are interested and concerned ... I value their interpersonal competence more than their theoretical expertise*.'	Participant 7, female, aged 51–60 years.
	What is normal?	*'For him* [the GP]*, as a man, I guess he is 45 years or something like that, things I have complained about, is really normal stuff. So, I have got feedback from my doctor that this, this isn’t something only you’re experiencing. Many people experience this. And I have known it. But still, it has been good to be comforted by my GP that this isn’t a crisis, then*.'	Participant 4, female, aged 41–50 years.
	The GP should see the whole picture	*' … she was really good at diseases. But it didn’t seem like she was comfortable with me digging into stuff like this. When that happened, she became quite detached*.'	Participant 8, female, aged 61–70 years.
GPs as biomedically competent life witnesses	The GP as a life witness	*'It is not okay when we are having a family party and he has been drinking on the quiet, you know. It is so embarrassing ... having couple relationship problems feels really lonely*.'	Participant 10, female, aged 61–70 years.
	In sickness and health, and through relationship problems	*' … I get lots of support* [from my GP] *to stay at a rehabilitation institution for a period of time to achieve more energy I can bring back to make my couple relationship work better. This support has been really helpful to me*.'	Participant 15, female, aged 61–70 years.

Holistic perspectives from biopsychosocial theory supported our analysis, providing a substantive theory foundation that enhanced our interpretive focus. Theory was not employed as a template framework, nor was it used to establish predetermined categories, nor was it a model to be empirically tested.

## Results

Eighteen participants from four different counties in Norway were interviewed regarding their experiences of discussing their couple relationships with their GP. The study covered 19 different GPs, as two participants had discussed their problems with three different GPs, and three GPs had been consulted by two different participants.

The participants were aged 32–66 years (median 45.5) and 72.2% were women. Almost half of the participants rated their health as poor (44.4%), and 61.1% had higher education. The characteristics of the participants are detailed in Table 3.

**Table 3.** Demographic variables of the 18 participants attending a qualitative interview study about patients’ experiences from talking with their regular GP about couple relationship problems (2021–2022)

Variables	*n*	Missing	%	Median	Minimum	Maximum
Age	18	0		45.5	32	66
*31–40 years*	*5*		*27.8*			
*41–50 years*	*6*		*33.3*			
*51–60 years*	*1*		*5.6*			
*61–70 years*	*6*		*33.3*			
Gender	18	0				
*Women*	*13*		*72.2*			
*Men*	*5*		*27.8*			
Urban/Rural	18	0				
*Urban*	*10*		*55.6*			
*Rural*	*8*		*44.4*			
Municipality	18	0				
*≥10 000 inhabitants*	*15*		*83.3*			
*<10 000 inhabitants*	*3*		*16.7*			
Self-rated health (Likert 1–5)	18	0		3	1	5
*Very good (4, 5)*	*5*		*27.8*			
*Good (3)*	*5*		*27.8*			
*Poor (1, 2)*	*8*		*44.4*			
Education	18	0				
*High school*	7		38.9			
*Higher education*	11		61.1			
Marital status	18	0				
*Married*	*9*		*50.0*			
*Cohabitant*	*3*		*16.7*			
*Divorced*	*5*		*27.8*			
*Widow(er*)	*1*		*5.6*			
Current marital/cohabitant time	18	0		16	2	45
*Not applicable*	*4*		*22.2*			
*<5 years*	*3*		*16.7*			
*5–20 years*	*4*		*22.2*			
>20 years	7		*38.9*			
RSS in current relationship	13	5		4.6	2.2	6.0
*RSS 5–6*	*5*					
*RSS <5*	*8*					
Experienced divorce/break-up?	18	0				
*No*	*7*		*38.9*			
*Yes, once*	*9*		*50.0*			
*Yes, twice*	*2*		*11.1*			
The consultation was about…	18	0				
…*a former relationship*	*7*					
*…the current relationship*	*11*					
The participant’s GP	17	1				
*Specialist*	*12*					
*In specialisation*	*5*					
The GP’s patient list size	17	1				
*>1000 patients*	*11*					
*≤1000 patients*	*6*					
The GP’s gender	16	3				
*Male*	*9*					
*Female*	*7*					

RSS = Relationship satisfaction scale.

The frequency of appointments addressing couple relationship themes varied, ranging from isolated consultations to regular meetings spanning several years.

Through the analysis process, three main themes emerged: 1) GPs in a facilitating role, not on an assembly line; 2) navigating the 'elephant in the room'; 3) GPs as biomedically competent life witnesses.

### GPs in a facilitating role, not on an assembly line

The participants described various aspects of the GP’s role that facilitated discussions about their couple relationship problems. Many perceived their GP almost as a signpost, providing them with information about other services:

'*Yes, the GP is a junction point, or... a place to go, and then they refer you to other specialists you need*.' (P10, female, 61–70 years)

Obtaining sick leave while experiencing couple relationship problems was seen by patients as an opportunity to address relational issues. They emphasised the important aspects of confidentiality and that general practice is a low-threshold service. For one participant, discussion with one GP also facilitated conversations about couple relationship problems with another GP:

'*It makes it easier for me to tell the other GP stuff regardless of if he asks about it. Because the first GP has asked me about it. It opens the door in a way.'* (P11, male, 31–40 years)

While the GP’s role does not usually encompass couples therapy, several participants described positive encounters when they had brought their partners to consultations. Involving a partner opened up new perspectives and enhanced the GP’s ability to uncover different ways of helping the patient.

Allocating sufficient time for discussion as well as the positive impact of continuity in a doctor-patient relationship in facilitating conversations were emphasised by all participants:

*'He’s not exactly young. So, it’s with dread that I've thought about perhaps soon having to replace my GP... The aspect of having continuity with one’s GP, I think, is quite important, especially if you're going to talk about personal matters.'* (P15, female, 61–70 years)

Beyond continuity and being familiar with their GP, trust was highlighted as another fundamental component enabling patients to discuss sensitive topics:

*'I think people trust their GPs, don’t they? That what they say is correct or wise, or that they know what they’re talking about*.' (P14, female 51–60 years)

Patients sometimes felt a sense of being rushed or treated like they were on a conveyor belt when the GP was running behind schedule:

*'I don’t know what the reason is that the GP is always too late, but I think there is potential for improvement … It reinforces the feeling of being a part of an assembly line*.' (P13, male, 31–40 years)

### Navigating the 'elephant in the room'

Patients valued GPs addressing couple relationship problems in consultations, thereby addressing what can be seen as the 'elephant in the room'. Several participants reported that they had initiated the discussion about couple relationship problems with their GP. They appreciated an engaged, supportive, and straightforward approach, which aligned with a biopsychosocial perspective. The participants described how this approach assisted them in viewing their problem from multiple perspectives, including their partner’s perspective. At the same time, it encouraged them to take accountability for their own situation, rather than feeling victimised. In some cases, the participant initially perceived the problem as solely somatic, but their GP asked about their relationship. These GPs’ holistic approach was appreciated:

' *… my GP had a direct approach, challenging the validity of my assessments and questioning my responsibility for the problems …* ' (P7, female, 51–60 years)

When GPs normalised couple relationship problems, participants found it easier to acknowledge that facing challenges is a normal aspect of any relationship. Conversely, some participants did not feel supported by their GP; rather, they described receiving advice that came across as arrogant and not fitting the situation. This left these patients struggling with challenges that they perceived as too difficult to overcome:

'*...kind of an arrogant type who doesn't have time for you... like you're practically thrown out headfirst before you even get a chance to open your mouth. So, then I think ... then it’s just pointless*.' (P12, female, 31–40 years)

Other participants had experienced a GP that did not want to talk about the 'elephant in the room' when they tried to initiate a discussion about couple relationship problems. Some participants longed for a more holistic approach from their GP, perceiving a narrow, one-dimensional biomedical focus. This failed to encompass their own understanding of the nature of their non-specific symptoms, such as sleeping problems, tiredness, or chronic pain:

' *… she was really good at diseases. But it didn’t seem like she was comfortable with me digging into stuff like this. When that happened, she became quite detached.'* (P8, female, 61–70 years)

Among the participants, varying opinions emerged regarding the extent of a GP’s personal involvement and experiences during a consultation. Some participants viewed the sharing of personal insights as an avenue to learn from the GP’s experience. One participant, however, felt that the GP’s personal experience reflected the patient’s narrative through the lens of her own story, resulting in a narrow, one-dimensional perspective:

'*Afterwards, it seemed like she* [the GP] *had experienced infidelity herself and judged me. It was not a good experience… It was hard to handle, and I felt I did not get the help I wanted… I think they should work hard to be neutral and... to put away their own* [perspective] *and be 100 % present in the consultation.'* (P6, female, 31–40 years)

### GPs as biomedically competent life witnesses

Several participants stressed the importance of having their GP act as a biomedically competent witness to their life events, particularly in instances involving what they described as shameful experiences. These could include instances of physical or mental abuse or having partners who struggle with addiction or gambling problems. Patients valued the attention that GPs gave to both relationship issues and medical concerns, as well as their ability to integrate these problems and help them make sense of their symptoms using a holistic perspective:

'*And when I came back again, I still talked about my stomach. But she was not so interested in that. She was interested in how things were for me at home.'* (P9, female, 41–50 years)

Topics such as infertility, infidelity, economic problems, living with a partner with a different sexual orientation, or experiencing sexual problems were also emphasised as shameful topics. Participants appreciated the opportunity to discuss these matters with their GP, even when they did not expect to receive direct solutions for their problems.

Patients expressed appreciation for the opportunity to discuss the impact of a chronic disease on their couple relationship. They valued talking with a professional who had knowledge about their disease and a capacity to comprehend relational matters. Older participants also highlighted the shame linked to experiencing couple relationship issues in older age and expressed how being able to share this perspective with their GP was beneficial:

'*And you feel that... Starting to have couple relationship problems when you are more than 60 years old, you know. But my GP made me feel not so strange*.' (P10, female, 61–70 years)

### Pathways in GP consultations leading to patients’ coping with couple relationship problems

**Figure 1.**
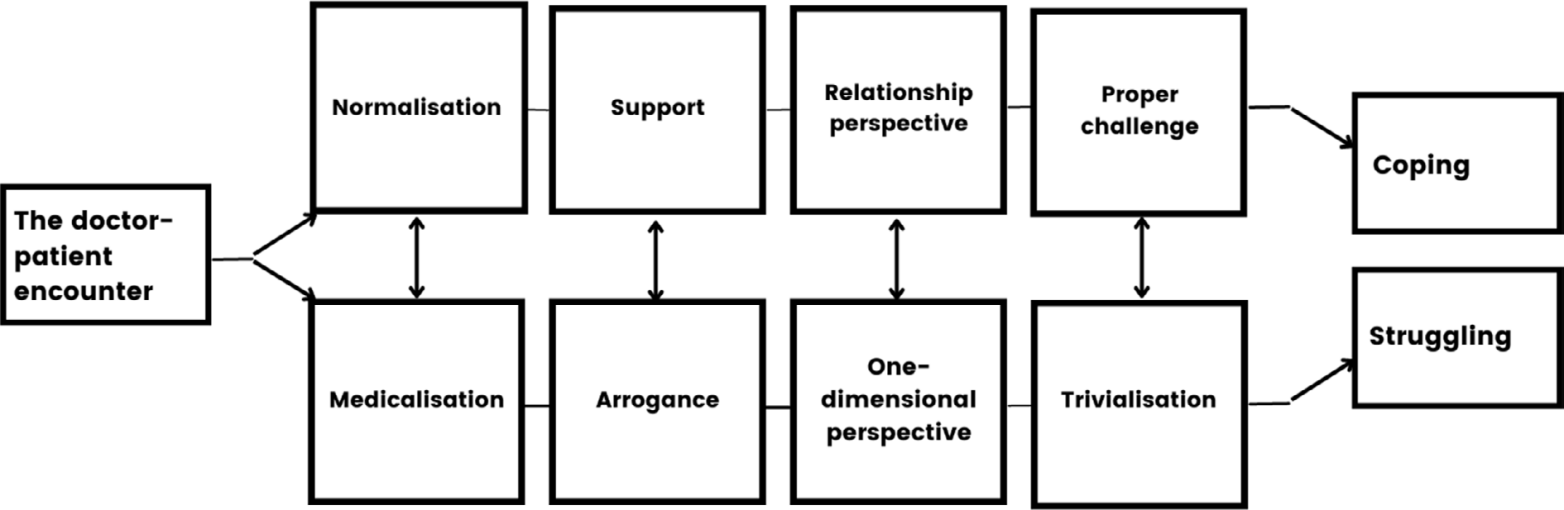
Pathways in GP consultations leading to patients’ coping with couple relationship problems.

Based on our data collected through the patient interviews, we developed a figure illustrating the components of both a positive and a negative consultation concerning couple relationship issues (Figure 1). This figure outlines pathways from *the doctor–patient encounter* to the patient’s experience of either *coping* or *struggling,* illustrating crucial elements along the way.

We have highlighted four elements in the proposed pathway to relationship coping: *normalisation, support, relationship perspective,* and *proper challenge,* all of which are grounded in our data. These elements are essential for the GP to be in position to help the patient cope with their situation. On the other side of the pathway, there are four opposite elements, also grounded in our data, to relationship struggling: *medicalisation, arrogance, one-dimensional perspective,* and *trivialisation*.

The element we have referred to as normalisation contains healing potential for the patient when experiencing the problems not as something very strange or special, but as something common and manageable. The opposite way of dealing with couple relationship problems would be the medicalisation of life burdens and associated symptoms.

In the support element, patients described feeling that the GP was on their team and that they had an ally in the process. In our data, when patients did not feel supported, they experienced the GP as arrogant.

To adopt a relationship coping perspective entails considering the patient’s affiliation with a social system, in this instance the couple relationship. Conversely, some patients described encountering a narrow, one-dimensional perspective from their doctor that did not contribute to making their situation more manageable.

A proper challenge emerges when the GP utilises a direct approach to motivate the patient to take personal responsibility for their situation. It is important to ensure that the level of challenge remains manageable for the patient. When the patients did not perceive this proper challenge, it led to a sense that the GP was trivialising their couple relationship problem.

Well-known factors important to a robust and professional doctor–patient encounter, such as trust, time, positive curiosity, and continuity, were emphasised by the participants. Conversely, factors like being part of an 'assembly line' and encountering various substitute GPs were identified as contributors to reinforce challenges within the relationship.

## Discussion

### Summary

The participating patients reported a varied frequency of discussing couple relationship problems with their GP, from one consultation to regular meetings over several years. We identified three main themes: 1) GPs in a facilitating role, not on an assembly line; 2) navigating the 'elephant in the room'; 3) GPs as biomedically competent life witnesses.

GP continuity was vital in fostering trust for discussing sensitive topics like relationship issues. Participants valued a biopsychosocial approach, incorporating close relationships into consultations about somatic issues. They appreciated both GP support and constructive challenges that prompted relationship improvements.

### Strengths and limitations

The internal validity of the study was strengthened by our constant referral to the interview transcripts during the analysis process, ensuring coverage for our conclusions. The conversations between the interviewer and each participant flowed easily whether conducted in person, by telephone, or digitally.^31^ While the interviewer used a semi-structured interview guide, it was not always necessary. Participants often brought in new perspectives, which led to different questions being asked to clarify the stories.

Awareness of the possible influence of the researchers’ own experiences and perspectives is important, and we aimed for reflexivity.^32^ All four authors are experienced GPs with a special interest in relational issues. The first author, who conducted the individual interviews, is educated in Gottman couples therapy.^33^ Two authors provide couple relationship education outside their GP practices. Two authors are educated in systemic therapy. Having a GP background is a strength when interviewing patients about GP consultations, as it aids in understanding the context and limitations of everyday clinical practice. However, there is also a risk of missing an outside perspective not provided by the participants.

A qualitative interview study is well-suited to address experiences of sensitive issues.^34^ We aimed to recruit a varied and adequate study sample, with a heterogeneous group in terms of gender, age, urban or rural location, relationship length, and different known risk factors for relational problems. To mitigate the risk of obtaining only success stories, we chose not to recruit patients through GPs. We encouraged them to contact the researchers via e-mail, which favoured individuals with a certain level of digital skill.

We did not have any gay participants. Only one participant had a non-Norwegian cultural background. The participants were of both genders, yet there was a majority of women. In retrospect, this could be related to the recruitment materials that primarily depicted white heterosexual patients. The recruitment process’ failure to yield equal numbers of male and female participants was a limitation of the study. Additionally, the lack of participants from sexual and gender identity minorities or migrant backgrounds further limited our findings.

The transferability of the findings to most GP patients was enhanced by a purposive sampling of participants, high information power, and reflexivity, which is crucial for external validity.^28^ Sample size determination utilised information power, taking into account factors such as narrow or broad study aim, dense or sparse sample specificity, applied established theory or not, strong or weak quality of dialogue, and case or cross-case analysis strategy.^28^ Our study aimed to explore patients’ experiences of discussing relationship problems in GP consultations, making it narrow in scope. The sample specificity was high due to the participants’ characteristics aligning closely with the study’s aim. Interviews featured strong dialogues with clear communication between the participants and the interviewer. We conducted an exploratory cross-case analysis, necessitating a larger participant pool than a case analysis with in-depth examination of narratives or details from a few selected participants.^28^

Our study concentrated on the sensitive topic of couple relationship problems. Thus, it was crucial to ensure participants’ well-being throughout the interviews, create a safe environment, and provide information on where additional help could be sought if desired. Data were securely stored, and quotations and stories were anonymised by the interviewer prior to being shared with the other authors.

### Comparison with existing literature

Most of our responders expressed appreciation for the GPs’ approach to addressing couple relationship problems during consultations. They emphasised how their GP showed an interest, offered support, and challenged them to take responsibility for their own situations. This resonates with the biopsychosocial theory of holistic care. Interest in and support of the patients’ couple relationships align with how psychosocial elements are an integrated part of people’s health, including the biomedical aspects. Furthermore, challenging patients to take responsibility for their own situations aligns with the model’s goal of empowerment. It encourages individuals to actively engage in their well-being, reflecting the behavioural aspects of biopsychosocial theory.^21,22,35^

Demonstrating acceptance towards patients is essential before challenging them. Bateson and Andersen advise a balanced challenge, one that is impactful but not overwhelming. They refer to this as 'a difference that makes a difference',^36^ in order to avoid perceived GP arrogance or detachment, as reported by some of our responders. This approach, which fosters change without being too burdensome, echoes recommendations for engaging with patients' emotional issues constructively.^9^

The patients we interviewed did not expect their GP to be a couples therapist. This aligns with the perspectives of doctors in other qualitative studies on addressing emotional concerns or couple relationship problems in consultations.^10,11^ GPs, while frequently engaging in discussions with patients on their couple relationships,^19^ did not see themselves as therapists.^11^

Some participants reported shame regarding late-life relationship issues, appreciating GP discussions on this sensitive topic. Younger participants anticipated therapist referrals from GPs for such matters. Norwegian GPs in another study observed limited referral options for older patients with relationship difficulties.^12^

Previous research shows patients are more likely to follow lifestyle advice or attend screenings when their GP is of the same gender.^37,38^ Our study’s limited scale does not allow us to conclude if this pattern also exists for relationship issues. Nevertheless, we did not reveal any trends related to gender in the patients’ consultation experiences.

A GP crisis is currently impacting several countries.^39–41^ The scope of tasks is expanding, yet appointment times are being constrained to accommodate these new demands.^42^ A pertinent question is whether GPs have enough time and should prioritise addressing relational problems and adverse events under resource constraints.

Couple relationship problems are intricate issues that show the interplay of biological, psychological, and social elements underscored by Engel’s biopsychosocial theory. These problems resonate with the model’s premise that health and well-being are influenced by a complex network of factors. Psychosocial stressors, such as those arising from couple relationship difficulties, can trigger physiological responses. These responses then impact prevalent diseases.^22^

Experienced GPs often discuss relationship issues with patients as part of holistic care. While some question their medical relevance, seeing them as life issues, they agree that if they impact health, they warrant attention. GPs seek tools to support patients when their health is affected by relationship troubles.^11^

McWhinney states that a GP prioritising adversities with a holistic approach to symptoms empowers the patient rather than medicalises patient distress.^30^ When time constraints force GPs into a biomedical, one-dimensional approach, it can harm patient well-being.^43^ Based on insights from Engel, McWhinney, and our findings, we posit that addressing life challenges in GP consultations normalises rather than medicalises patients’ experiences.^22,30^ This approach empowers patients to manage their distress and mitigates overdiagnosis and overtreatment.

### Implications for research and practice

Further research should focus on the testing and validation of straightforward tools that could prove valuable in general practice. These tools might encompass easily accessible digital resources aimed at enhancing couple relationships, tools that GPs can employ during consultations to facilitate productive discussions about couple relationship issues, or assessment tools designed to simplify the process of evaluating such problems.

GPs should be aware that relationship issues may be associated with medical conditions. Normalising these difficulties, validating patient emotions and experiences, and helping them understand their partner’s perspective are key. Constructive challenges can foster positive changes, aiding in relationship management, benefiting overall health, and minimising overdiagnosis and overtreatment.

Patients value their GPs’ holistic, supportive, and direct approach in addressing couple relationship problems, although they perceive that GPs do not always have sufficient time. They welcome relevant challenges that can drive positive change.

## Data

The data underlying this article cannot be shared publicly for ethical reasons and the privacy of the individuals who participated in the study. The data will be shared on reasonable request to the corresponding author.
